# PrestOMIC, an open source application for dissemination of proteomic datasets by individual laboratories

**DOI:** 10.1186/1477-5956-5-8

**Published:** 2007-06-06

**Authors:** Charles G Howes, Leonard J Foster

**Affiliations:** 1 UBC Centre for Proteomics, Department of Biochemistry and Molecular Biology, University of British Columbia, Vancouver, BC, V6T 1Z4, Canada; 2 Packets Consulting, Inc., Burnaby, BC, V6T 1Z4, Canada

## Abstract

**Background:**

Technological advances in mass spectrometry and other detection methods are leading to larger and larger proteomics datasets. However, when papers describing such information are published the enormous volume of data can typically only be provided as supplementary data in a tabular form through the journal website. Several journals in the proteomics field, together with the Human Proteome Organization's (HUPO) Proteomics Standards Initiative and institutions such as the Institute for Systems Biology are working towards standardizing the reporting of proteomics data, but just defining standards is only a means towards an end for sharing data. Data repositories such as ProteomeCommons.org and the Open Proteomics Database allow for public access to proteomics data but provide little, if any, interpretation.

**Results & conclusion:**

Here we describe PrestOMIC, an open source application for storing mass spectrometry-based proteomic data in a relational database and for providing a user-friendly, searchable and customizable browser interface to share one's data with the scientific community. The underlying database and all associated applications are built on other existing open source tools, allowing PrestOMIC to be modified as the data standards evolve. We then use PrestOMIC to present a recently published dataset from our group through our website.

## Background

Mass spectrometry (MS)-based proteomic methods have developed to the point where many laboratories can routinely identify hundreds or thousands of proteins from complex samples such as plasma, biochemically enriched organelles, isolated protein complexes and whole cell lysates [1-3]. The results from early, small-scale 'proteomic' studies could be and were presented entirely within a standard journal article, even including spectral assignments. However, the standard journal article format is not a useful medium to present the raw data of the much larger datasets being generated today or, indeed, enormous lists of data of any kind. As an example in a related field, when a sequenced genome is 'published' it does not imply that the journal actually prints the hundreds of millions or billions of base pairs comprising the genetic blueprint for the organism. In the case of such studies the journal article focuses on the interpretation of the sequence data while the actual sequence is deposited into public databases maintained by the National Center for Biotechnology Information (NCBI), the European Bioinformatics Institute (EBI) or other institutions. Similar public databases are available for microarray data [4-6] and databases for proteomics data are starting to emerge [7-11]. However, these databases are intended more as large public repositories of raw and/or interpreted data rather than value-added presentations of specific datasets; in such large repositories the message and individuality of each of the research projects is lost.

The incompatibility between the standard journal article format and large-scale proteomics datasets means that the true value of such work is often not realized by the scientific community because much of the data is packed away in long, mundane tables of accession numbers. Such data could be better presented through a specifically designed web portal, and while no journal yet requires such a presentation, a few larger laboratories have started to develop websites for specific projects [12-14]. This is often beyond the capabilities of many groups however, so here we describe PrestOMIC (*presto *is Latin for 'display'): a simple suite of tools for creating a customizable database for sharing liquid chromatography/tandem mass spectrometry (LC/MS)-based proteomic data.

## Results

### Underlying database

**Figure 1:**
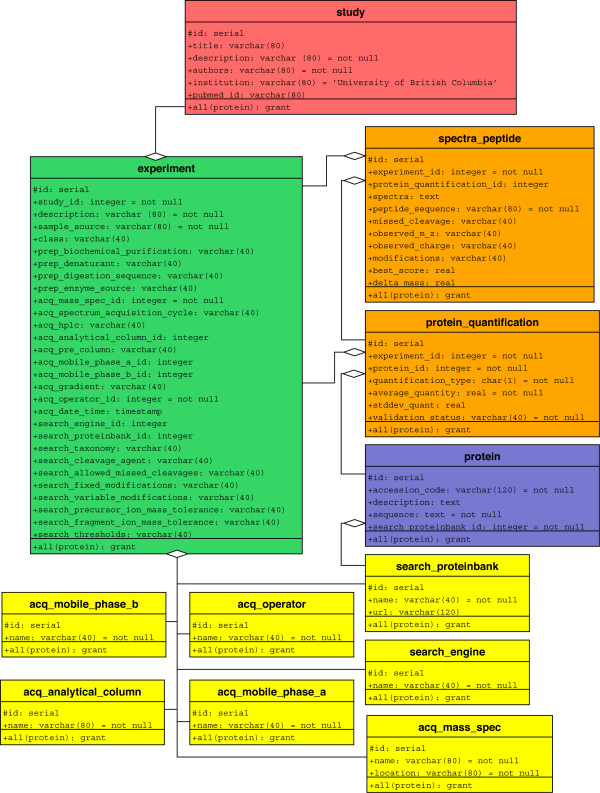
**PrestOMIC database schema**. Schematic representation of relationships among tables in PrestOMIC. The *Study *table represents the highest level in the table. Relationships between tables are indicated by lines. All relationships are 'one to many', with the 'many' end indicated by the open diamond (e.g., many *Experiments *can be associated with one *Study*). The acquisition and search parameter tables in yellow at the bottom all have a one-to-many relationship with *Experiment*.

The microarray community agreed on the MIAME (Minimum Information About a Microarray Experiment) standards in 2001 [15] but the proteomics community is still far from an agreement on an analogous MIAPE despite the best efforts of the Human Proteome Organization's (HUPO) Proteomics Standards Initiative (HUPO-PSI) [16]. Our aim here was not to build a database capable of integrating enormous datasets from disparate groups but rather to construct a system that an individual research group could use to present their data through their own website. Optimally such a system should be completely open and non-proprietary to encourage as many users as possible, it should be powerful enough to easily handle datasets containing thousands of protein entries and it should adhere as much as possible to emerging guidelines for presentation of such data. To this end the underlying relational database used in PrestOMIC was constructed in PostgreSQL [17] according to the schema depicted in Figure 1.

The intent was to create a forum for a group to share data from different projects within their laboratory, so the top-level unit here is the '*Study*', a table which holds some general information about each project, including the title, authors and PubMed identification number (when referring to the name of a table in the PrestOMIC schema we have capitalized the first letter and italicized the word). Each *Study *entry could have several different experiments within it (e.g., biological replicates, technical replicates, similar analyses on different sources, similar analyses on different mass spectrometers, etc.) so the *Experiment *table contains all the relevant information about the sample and analysis of that sample. Specifics about the mass spectrometry configuration, analytical column, mobile phases and search engine are stored in separate tables within the schema to avoid unnecessary replication and referred to by their respective identification numbers in the *Experiment *table. At the time of writing the standardized vocabulary for describing many of these parameters was still under development at HUPO PSI [16] so the data is stored as uncontrolled strings at this point.

In a typical proteomics study the central dataset is the list of proteins the authors are claiming to have identified. Layered on top of this list might be relative or absolute quantitative data, post-translational modifications, or other information, but the central pieces of data are still the proteins, their accession numbers and their amino acid sequences. As such, we elected to create a separate table called *Protein *to hold all the protein sequences identified in a *Study *entry, again to avoid replication, since a protein could be identified in several *Experiments *within a *Study*. The *Protein_Quantification *table, as the name suggests, contains the quantitative information measured for a protein, if any exists. Since the quantitative measure of a particular protein sequence could be different in different *Experiments*, one-to-many relationships link *Protein *and *Experiment *to *Protein_Quantification *(Figure 1). In the case where an *Experiment *is only qualitative, each protein identified has an entry in the *Protein_Quantification *table but the fields pertaining to quantitation are left blank. Finally, the *Spectra_Peptide *table holds all the data about the individual peptides used to identify and quantify these proteins, including a field for the tandem mass spectra used in the identification.

Proteins are the primary output of a MS-based proteomics experiment but their identification is dependent on the acquisition and analysis of mass spectra: typically tandem mass spectra, but sometimes peptide mass fingerprints. Some journals now encourage authors to make the underlying spectra used for protein identification available [18] but there are as yet no effective tools for this task. Optimally such a tool would use a universally recognized standard for describing a mass spectrum but the HUPO-PSI has not yet decided on this format. Since most protein identification search engines accept files in the so-called Mascot Generic Format (MGF) we decided to base our spectrum display on this format for the time being and then to adapt PrestOMIC once a common format is agreed upon.

### Installation and data entry

PrestOMIC requires two major underlying applications, a web server and a relational database system. Because of their stability, popularity and open source nature, we have chosen to use the Apache web server (v2.2.2) and PostgreSQL (v8.1.4) installed on a single processor computer running the Fedora Core 5 version of Linux (see Methods for detailed configuration information). The database schema can be entered manually based on Figure 1 or more efficiently by importing the schema file available as Supplementary Material or from the PrestOMIC Subversion system on Google [19]. Since one installation of PrestOMIC can handle several different projects, a user should create a top-level hypertext markup language (HTML) page to link to the individual projects and then organize all the files for each project within its own directory. Several aspects of the main page for each project can and should be customized: 1) The title banner, 2) Text describing the project, 3) Any Venn diagrams or other project-specific graphics, 4) Conditional statements for differential searches (see description of browser interface below).

Once the system is configured, data for the project can be entered into the database via two mechanisms: manually through a program called phpPgAdmin (v4.1), or in batch mode as a CSV-formatted text file using the import functions in PrestOMIC. For the high-level tables we find it easier to enter the data manually, but for protein and peptide information it is more efficient to use the import functions in PrestOMIC. These may be accessed through the command line, and as long as the comma-separated table is formatted correctly (see Methods for the required format) then the project will be loaded correctly.

### Browser interface

The browser interface for PrestOMIC is the portal through which interested outside users may view or query the data being presented. Our aim in designing this interface was not to build in all possible bells and whistles an investigator might want to use to make a flashy website, but rather to provide simple, searchable access to the data. As such, the main page for each project is intended to contain basic information about the project, including: title, authors, link to journal website once published, a description of the project such as the manuscript abstract (if the publishing journal's policies allow it), and perhaps a graphical representation of the study. In our first practical use of PrestOMIC we have also added links so a reader can download the supplementary data files (see below).

**Figure 2:**
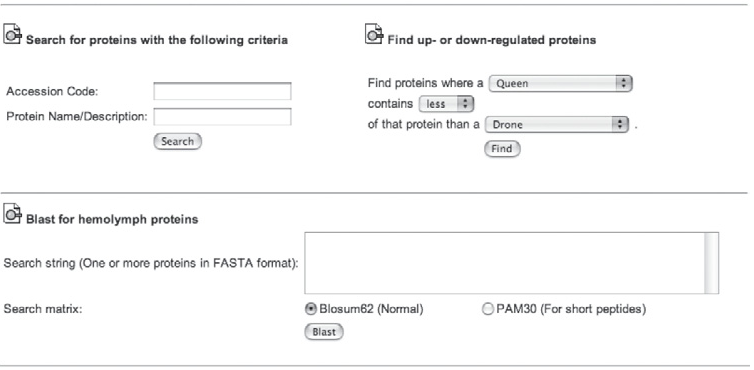
**PrestOMIC search functions**. Partial screenshot of the general search functions provided in PrestOMIC. The Protein table can be queried by accession number, protein description or sequence (via BLAST). The *Protein_Quantification *table can be queried by relative abundances between two different *Experiments*.

The main PrestOMIC page then provides numerous tools for querying the database: 1) Graphic maps: One common approach in proteomics is to analyze samples from several distinct sources (e.g., different organelles, different tissues, etc.) so PrestOMIC allows for user-defined graphics which, if image-mapped (see Methods), can be used to retrieve the appropriate data (see Application below). 2) Accession number or protein description search: Two fields are provided to allow an interested party to search the *Protein *table by accession number or name. These searches are generally applicable to all types of data and so do not require study-specific modification (Figure 2). 3) Conditional searches: A common goal in proteomic studies is to identify differentially expressed proteins (e.g., proteins more abundant in sample A than in sample B) so PrestOMIC allows conditional searches of the *Protein_Quantification *table (Figure 2). This kind of conditional search is obviously dependent on how a particular study was performed so the conditional statements need to be changed as appropriate (see Methods). 4) BLAST: Perhaps the most useful search function gives a user the ability to BLAST [20] his/her sequence of interest against the entire set of proteins identified in a study, a function which is also generally applicable (Figure 2). The function will return the entire BLAST output, linking each hit to the entry in PrestOMIC, so the user can decide what level of homology s/he wants to accept.

**Figure 3:**
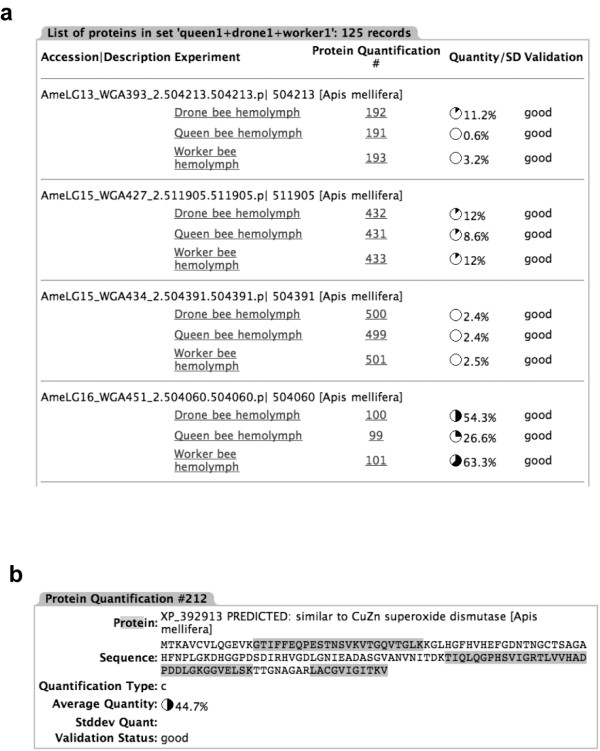
**Data views**. a) Partial screenshot of the protein list view in PrestOMIC. The *Experiment *each of the proteins was found in is listed, as is the unique identification number for the protein in the *Protein_Quantification *table, the relative quantification of each protein (in this case sequence coverage was used) and the validation status of each protein. Most proteins reported would obviously be validated, but an investigator might also want to report contaminants, for instance, and that status could be indicated in this last column. Clicking on the hyperlinked *Experiment *retrieves all the peptides identified in that *Experiment *(not shown) and clicking on the hyperlinked. *Protein_Quantification *number brings up b) the information specific to that protein in that particular *Experiment*. Each peptide identified is highlighted and hyperlinked. Clicking on the link brings up additional information about the specific peptide, if it is available, such as the observed mass-to-charge ratio (m/z), the observed charge state(s), modifications and the fragment spectra used in the identification.

**Figure 4:**
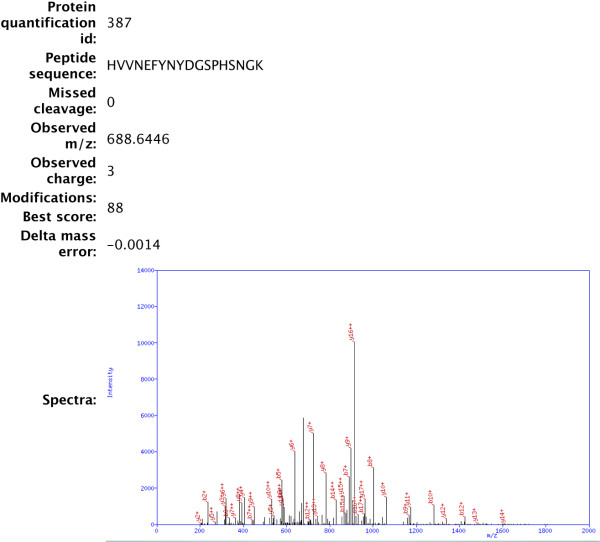
**Display of peptide information and fragment spectra**. Partial screenshot of the information available for an individual peptide within PrestOMIC. The amino acid sequence, number of missed cleavages, observed m/z, observed charge, modifications, database score and delta mass error in parts-per-million. In addition, PrestOMIC has the ability to display and annotate peptide fragment spectra.

Any retrieval query from the main page will bring up a page listing all proteins matching the query and the different *Experiment *entries they were identified in, providing links to their accession numbers, unique identification number within the *Protein_Quantification *table, and relative quantity measured in each *Experiment *(Figure 3a). Clicking on the hyperlinked 'Protein Quantification #' takes a user to a view of the protein and the information gathered for it in that particular experiment (Figure 3b), including a view of the protein sequence and the locations of sequenced peptides. Clicking on the linked experiments retrieves all data for that particular experiment, including all the recorded data acquisition parameters and all the peptides. The pertinent information for each peptide is also available, including the sequence, precursor ion m/z, measurement error and database score, in addition to an annotated fragment spectra (Figure 4).

### Application of PrestOMIC to a real-world dataset

**Figure 5:**
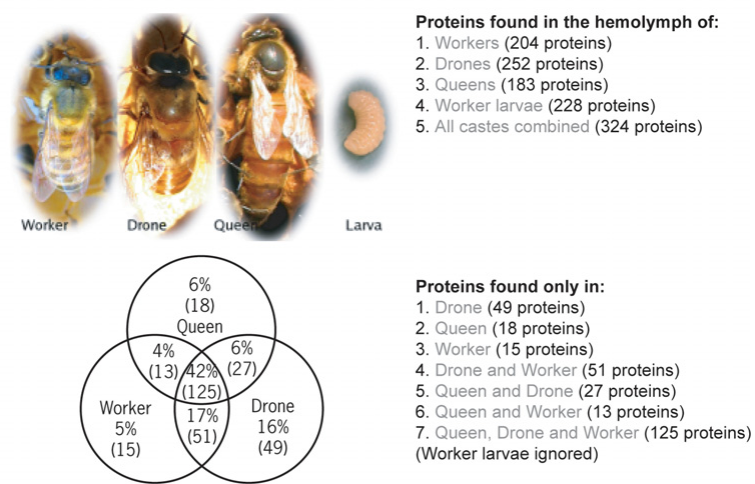
**PrestOMIC customized to present honeybee hemolymph data**. Partial screenshot of the homepage for our honeybee hemolymph project [22]. The worker, drone, queen and larva images are hyperlinks that will retrieve all the proteins associated with each caste, as are the light grey words in the table to their right. The Venn diagram depicts the overlap between each of the adult castes and clicking in any sector of the diagram will retrieve the appropriate subset of data.

We have used PrestOMIC through our group's website [21] to present the data from a recent study in our laboratory where we examined the composition of honeybee hemolymph [22]. From the main project page a user can retrieve all the proteins identified from each life stage or caste, the intersecting set of proteins between any combination of the three adult castes (Figure 5) and a semiquantitative estimate of those proteins more abundant in one caste versus another (Figure 2). The protein description, accession number and BLAST search fields described above are also enabled.

## Discussion & conclusion

The need for tools such as PrestOMIC stems from the incompatibility between a standard journal article and the very large datasets being generated in proteomics. It is neither economically viable for journal publishers to print pages and pages of tables, nor is it interesting for their readers, so typically such information is made available electronically through journal websites. This is a useful format for investigators who wish to reanalyze the data but it is cumbersome and not very accessible for someone who, for instance, works on a specific protein and wants to know if the authors found that protein in the particular conditions. We have also found from experience that it can be very challenging to effectively review such material when it is submitted to a journal. Therefore, we have created PrestOMIC primarily as a presentation aid for such datasets.

The scalability of a PostgreSQL database ensures that PrestOMIC should be able to handle any conceivable dataset generated in proteomics since there are a finite number of possible gene products. Even so, we do foresee at least two upgrades to PrestOMIC that will be required in the near future but that cannot be implemented at this time. As mentioned, there are still no agreed-upon standards for proteomic data [23] but once HUPO-PSI publishes standard vocabularies and spectrum descriptions they will be implemented in PrestOMIC. However, given how long it took MIAME standards to develop, and even now they are not universal, it seems prudent to make PrestOMIC available in a perhaps immature form now rather than wait several years for a 'perfect' version. We will incorporate these upgrades ourselves but the PrestOMIC code is available on Google's SubVersion system [19] so we also encourage community contributions to PrestOMIC's development as well.

While PrestOMIC will have some catching up to do in the future, many data pipelines in proteomics will also need to be upgraded to fully utilize PrestOMIC. In the larger picture, the pipelines will have to be upgrade to even satisfy the publishing requirements that are inevitably coming. For instance, most data pipelines break the link between raw fragment spectra and peptide early on so that at the time of publication it is virtually impossible to go back and gather all those spectra that gave rise to the identifications. We are currently re-engineering our own pipeline to address this.

PrestOMIC is not the first system created for presenting proteomic data [12-14] but to the best of our knowledge it is the first where the structure itself is available to the wider scientific community. While publishing standards in proteomics have been slow to emerge it is clear that some form of public presentation of the data will likely be required [18]. PrestOMIC, by providing customizable tools for a compact and interactive presentation of specific datasets, will allow investigators to increase the exposure and impact of their data, benefiting themselves and the publishing journal alike.

## Methods

### Installation and configuration of PrestOMIC

Perl, the Apache Webserver and PostreSQL are installed as part of Fedora Core 5; if a different operating system is used then Apache [24] and PostreSQL [17] must be installed from their respective websites. Additional Perl libraries need to be installed, primarily BioPerl [25], CGI::FormBuilder [26] and Template Toolkit [26]. The schema file available from Supplementary material, the PrestOMIC project website [19] or our own website [27] can then be imported into PostgreSQL to configure the database. Finally, the webpage files are copied into an Apache-accessible directory and customized (see below) as needed.

### Customization of the PrestOMIC main page

There are four main aspects to the main HTML page that can be modified:

1) The title banner – the current dbtitle.jpg file in /images/ can be replaced with a graphic symbolic of the study being presented.

2) Text describing the project – text specific to the project is scattered throughout index.html and should be changed to suit the need. These include: page title, meta content and information about the journal article.

3) Project-specific graphics – in the sample dataset used we have incorporated Venn diagrams to demonstrate overlap between different hemolymph samples and images of each of the different castes and life-stages. Again, depending on the data being presented, different image-mapped, hyperlinked graphics should be used here. In order to increase security and to prevent outside users from passing SQL statements directly to the server (an SQL injection attack), the hyperlinks to retrieve the union or intersection of datasets pass an argument containing the *Experiment *name plus a boolean value of 1 or 0 indicating whether the data from that *Experiment *is to be included in the query. For example, the Proteins Found Only In Drone and Worker link [28] is translated by PrestOMIC into a series of SQL statements that determine the requested subset of proteins. This example looks in our four-class database (queen, worker, drone, larva) for proteins that are present in both workers and drones, and are absent in queens. The list of proteins present in larvae has no effect on this query, because 'larva' did not appear in the query. This code is generalized, and will not need to be modified if more classes are added; the words in the query are from the 'class' field of the experiments of interest. The '1' or '0' appended to each word indicate whether a protein must be present in the class or absent from the class, respectively, before it can be included in the subset. A search limited to two classes (say 'queen' and 'worker') can be represented by a two-set Venn diagram (queen and worker, not queen and not worker, queen and not worker, not queen and worker) and can co-exist in the same database with all other possible Venn diagram searches. Any number of classes can be represented by a Venn diagram for the corresponding number of sets, but while two-dimensional Venn diagrams with more than four sets can be generated with some straightforward rules, they are difficult to draw, more difficult to turn into an image map, and even more troublesome to actually interpret.

4) Conditional search statements – to change the subjects of the conditional searches the values in the 'option' tags of the table containing the 'regulation search' need to be changed to match the values in the *Experiment *table of the database for the particular project.

### Open source code

The Perl code for PrestOMIC is maintained in the Google SubVersion system [19] and can also be downloaded from our website [27] or from ProteomeCommons [11]. The HTML template for the main page and the database schema in SQL format (PostgreSQL flavour) are also available at each of these sites.

### Data entry

As mentioned above, all data can be entered manually if desired, but for protein and peptide data in particular it is far more efficient to simply upload a file containing all the data. Additional File 3 in the supplementary data contains the expected format for such data. To enter the data into the database, transfer the file onto the server and run the command 'addstudy < studyfile.csv'. To backup the data from the database, run the command 'dumpstudy 123 > study123.csv', where the number is the study number. To delete the study from the database, run the command 'delstudy 123', where the number is the study number. If PrestOMIC is moved to a different SQL database, 'delstudy' will need to be expanded, as it's heavily dependent on PostgreSQL's 'cascade delete' feature to erase all records pertaining to a study.

## Competing interests

CGH owns Packets Consulting, Inc., an internet consulting business.

## Authors' contributions

CGH authored all the code developed here and implemented the whole project. LJF conceived of the idea for PrestOMIC, developed an initial early version of the schema, directed the development of the database and the interface, and wrote the initial draft of the manuscript. Both authors read and approved the final manuscript.

## Supplementary Material

Additional File 1 **PrestOMIC database schema**. This file is a dump of the schema from PostgreSQL and can be read directly back into PostgreSQL or any other relational database application that handles SQL, with some pruning of PostgreSQL-only statements. It can also be viewed in a standard text editor. Click here for file

Additional File 2 **PrestOMIC HTML page**. An HTML template page for constructing a main page for a PrestOMIC project. It may be viewed in a web browser but should be edited in a general text or HTML editor. Click here for file

Additional File 3 **Sample input data file**. A sample data file demonstrating the expected format for automated input into PrestOMIC. Click here for file
