# Supplementary material for: PrestOMIC, an open source application for dissemination of proteomic datasets by individual laboratories
Source: Proteome Sci. 2007 Jun 6;5:8. doi: 10.1186/1477-5956-5-8 (PMC1892544; doi:10.1186/1477-5956-5-8)
Supplement: Additional File 2 — PrestOMIC HTML page. An HTML template page for constructing a main page for a PrestOMIC project. It may be viewed in a web browser but should be edited in a general text or HTML editor. [file 1477-5956-5-8-S2.html]

Honeybee Hemolymph Protein Database


|  |  |  |  |  |  |  |  |  |  |  |  |  |  |  |  |  |  |  |  |  |  |  |  |  |  |  |  |  |  |  |  |  |  |  |  |  |  |  |  |  |  |  |  |  |  |  |  |  |  |  |  |  |  |  |  |  |  |  |  |  |  |  |  |  |  |  |  |  |  |  |  |  |  |  |  |  |
| --- | --- | --- | --- | --- | --- | --- | --- | --- | --- | --- | --- | --- | --- | --- | --- | --- | --- | --- | --- | --- | --- | --- | --- | --- | --- | --- | --- | --- | --- | --- | --- | --- | --- | --- | --- | --- | --- | --- | --- | --- | --- | --- | --- | --- | --- | --- | --- | --- | --- | --- | --- | --- | --- | --- | --- | --- | --- | --- | --- | --- | --- | --- | --- | --- | --- | --- | --- | --- | --- | --- | --- | --- | --- | --- | --- | --- |
| |  | | --- | | *Image courtesy of DHD Multimedia gallery* | | --- | | | | | | | | Welcome to the Honeybee Hemolymph Proteomics Database at the University of British Columbia.    This database accompanies the article: Quantitative comparison of caste differences in honeybee hemolymphby Queenie W.T. Chan†, Charles G. Howes†, and Leonard J. Foster†°  **†** UBC Centre for Proteomics, Department of Biochemistry and Molecular Biology, University of British Columbia, Vancouver, BC V6T 1Z4.  ° Corresponding author, e-mail: , Telephone: +604-822-8311, Fax: +604-822-2114 Abstract The honeybee, *Apis mellifera*, is an invaluable partner in agriculture around the world, both for its production of honey and, more importantly, for its role in pollination. Honeybees are largely unexplored at the molecular level, despite a long and distinguished career as a model organism for understanding social behavior. Like other eusocial insects, honeybees can be divided into several castes: the queen (fertile female), workers (sterile females) and drones (males). Each caste has different energy and metabolic requirements and each differs in its susceptibility to pathogens, many of which have evolved to take advantage of the close social network inside a colony. Hemolymph, arthropods' equivalent to blood, distributes nutrients throughout the bee and the immune components contained within it form one of the primary lines of defense against invading microorganisms. In this study we have applied qualitative and quantitative proteomics to gain a better understanding of honeybee hemolymph and how it varies amongst the castes and during development. We found unexpectedly large differences in hemolymph protein composition, especially between larval and adult stage bees and between male and female castes but even between adult workers and queens. We also provide experimental evidence for the expression of several unannotated honeybee genes and for the detection of biomarkers of a viral infection. Our data provide an initial molecular picture of honeybee hemolymph, to a greater depth than previous studies in other insects, and will pave the way for future biochemical studies of innate immunity in this animal.   |  |  | | --- | --- | |  | **Proteins found in the hemolymph of:**  1. Workers (204 proteins)  2. Drones (252 proteins)  3. Queens (183 proteins)  4. Worker Larvae (228 proteins)  5. All castes combined (324 proteins) |  ---  |  | **Proteins found only in:**  1. Drone (49 proteins)  2. Queen (18 proteins)  3. Worker (15 proteins)  4. Drone and Worker (51 proteins)  5. Queen and Drone (27 proteins)  6. Queen and Worker (13 proteins)  7. Queen, Drone, and Worker (125 proteins)  (Worker larvae ignored) | | | | | | | | | ---  Download the experimental data Protein quantification data (OpenOffice Calc)   Protein quantification data (Excel)   Protein quantification data (CSV)   Proteins found in Apis hemolymph (Fasta) | | | | | | | | |  |  | | --- | --- | | --- | | | Last updated 2006-10-15 | Database released, 2006-05-01 | | Website and database designed/maintained by Charles Howes | | | | | | | | |
